# A Physician's Apologia

**Published:** 1890-06-14

**Authors:** 


### The Doctor's Pulpit.

### A PHYSICIAN'S APOLOGIA.

All Great Britain was informed the other day, through the daily Press, that the President of the Royal College of Physicians of London had made a public and definite avowal of his belief in Christianity. There was a time when such an avowal by a president of the College of Physicians would have been of little moment to the scientific world, because the occupancy of the presidential chair did not always imply a profound familiarity with the thoughts and ways of science. The case is different now. Any man who is entrusted with the presidency of any British College of Physicians is *ipso facto* demonstrated to be a scientific man. Sir Andrew Clark is, however, not a mere undistinguished subaltern in the army of scientists. He has had conferred upon him almost every honour that English, Scotch, or Irish universities, Royal colleges, and Royal societies have it in their power to give. He differs from the mere text-book and museum scientist exactly as Wellington differed from a colonel of militia. He has been in the thick of the actual fight for forty years. The record of his purely scientific work shows at a glance the practical and strenuous nature of his experience.

The story of the life-conflict of a man like Sir Andrew Clark, if given to the world in perfect simplicity and fulness of detail, would probably prove as interesting as the Confessions of Augustine, the Apologia of J. H. Newman, or the world-denunciations and unconscious self-revelations of Carlyle. But medical men do not indulge in autobiographies, or even in the mild enjoyment of personal diaries. Let us hope that an eminent physician may yet be found who will accept the mantle of Samuel Pepys, and interweave with it that of Saint Augustine. Failing this, some Boswell might seek out a medical Johnson, and make known his thoughts and doings to the world. The actual inner life and professional experience of an able and honest physician would furnish intellectual food of a rare and diversified kind.

Sir Andrew Clark says he has passed through "seas of doubt." That is a familiar experience. In these days intellectual uncertainty has to a large extent taken the place of gross animal desires as a form of temptation. Before the Renaissance men did not so much question the theory of the universe presented to them in the Old and New Testaments, and on the authority of the Church, as they felt the practical difficulties of resisting their own coarser passions and bringing their lives into actual conformity with the theory. In these days the standard of taste and the necessity for professional success have combined to put an effectual check upon the passions; and if men are to be tempted at all they must be tempted by other means. That men are tempted, that life is what Carlyle called a "hand-to-hand grip with destiny"—a conflict sometimes so dire that a successful issue for the human wrestler seems to be impossible—no man of forty will deny. Intellectual doubt is the antagonist that assails an intellectual man. But to a man of the highest and strongest intellectual type the vanquishment of doubt is as imperative a necessity as the overcoming of any other difficulty that stands in his way. Why has Sir Andrew Clark arrived at what he calls the "quiet haven" of definite belief? Those who know him will understand that one reason is the intense definiteness of his intellectual character. It is not possible for him to coast about for ever on a sea of uncertainty; he must land somewhere. And so must every man of the highest type of mind.

There are some very eminent persons who have rendered great service to physical science, and others who have at least striven to further the cause of social and political science, who in the highest moral and intellectual spheres proclaim themselves "Agnostics." However philosophical agnosticism may be as a state of mind, it has the suspicious peculiarity of being entirely unpractical. In all the common departments of life it is necessary to make up one's mind one way or another. In getting out of bed in the morning a man must put on his stockings, or he must leave them off—one or the other. He cannot sit all day long on the edge of the bed in his night-gown in an agnostic condition. So when he goes down to breakfast he must choose either the bacon or the eggs, the fish or the devilled kidney; or he must select both bacon and eggs or some other combination; or he must do his best to try the flavour of them all. What he cannot do is to sit at table in a purely speculative attitude and take nothing. So in the choice of a wife, decision, one way or another, is absolutely essential. Mary has many points to recommend her; Jane is both sweet and beautiful; Eliza has a thousand a-year in her own right, and her mother has been sent upon a diplomatic mission to the Cannibal Islands; and so on through the whole gamut of charming personalities endowed with feminine Christian names. But whilst it is possible to choose Mary, or Jane, or Eliza—or, in a Mormon country, all three of them—what is not possible is to have a wife without making up one's mind about her. Agnosticism may be, and in many cases certainly is, a very honest state of the intellect; but undoubtedly it is as feeble as it is honest. In ordinary affairs the want of decision of character invariably leads to one, and but one result—practical failure. It is not likely that in the higher regions of thought indecision can be more meritorious than in the lower, or can lead to any more desirable consequences.

Sir Andrew Clark considers that Christianity appeals both to the head and to the heart. He says in effect what Paley and so many others have said before him. There are many things in the world, some the work of nature, and others the work of man. He can get to the bottom of some of these, but others he finds are beyond him in more or fewer particulars. But all those he can get to the bottom of he knows to be the work of intelligence and purpose, and all the intelligence and purpose which he has ever met with he has found associated with personality and individuality. Hence he concludes, and that with the most absolute scientific warrant, that things which he cannot get to the bottom of, but which unmistakeably show purpose and intelligence, have likewise been associated in their origins with personality and individuality. In other words, as we cannot have a watch without a watchmaker, so neither can we have a world without a world-maker.

The settled convictions of Sir Andrew Clark include this among them, that Almighty God has, for practical purposes, made Himself seen and understood of men by means of Jesus Christ. Sir Andrew thinks, as most men think who have done all that was possible for them to elucidate the subject, that Christ, with all that is connoted by His name, was the meeting of a practical necessity of man. The intellectual and moral illuminations and forces that came in with Christ were as necessary for the higher development of man at that stage of his history as solid food is for the boy who has ceased to be an infant, or as philosophy is for the student who has mastered the rudimentary facts of life and man. It is impossible in the space at our disposal to do more than state this thesis. But we may be permitted to add a word by way of conclusion. Carlyle assures us that one man's convictions are immeasurably strengthened by the similar convictions of another man. We feel both encouraged and grateful, and so will many more, especially among young men, be encouraged and grateful, for the steadfast attitude of Sir Andrew Clark in the face of the profoundest of all problems that modern civilised man is called upon to solve.

